# Intestinal Flora Changes Induced by a High-Fat Diet Promote Activation of Primordial Follicles through Macrophage Infiltration and Inflammatory Factor Secretion in Mouse Ovaries

**DOI:** 10.3390/ijms23094797

**Published:** 2022-04-27

**Authors:** Zhihao Fan, Xiaoqian Zhang, Yanxing Shang, Maosheng Zou, Meng Zhou, Qiukai E, Shujia Fei, Wei Chen, Jing Li, Xuesen Zhang, Xiaoqiu Liu

**Affiliations:** 1 State Key Laboratory of Reproductive Medicine, Nanjing Medical University, Nanjing 211166, China; fanzhihaokeyan@126.com (Z.F.); zhangxq@njmu.edu.cn (X.Z.); syx934619964@163.com (Y.S.); zoumaosheng163@163.com (M.Z.); zhouM0607@pumcderm.cams.cn (M.Z.); redlion1986@163.com (Q.E.); shujiafei15@163.com (S.F.); chenw0321@163.com (W.C.); ljwth@njmu.edu.cn (J.L.); 2 Key Laboratory of Human Functional Genomics of Jiangsu Province, Department of Biochemistry and Molecular Biology, Nanjing Medical University, Nanjing 211166, China; 3 Key Laboratory of Pathogen Biology of Jiangsu Province, Department of Microbiology, Nanjing Medical University, Nanjing 211166, China

**Keywords:** high-fat diet, intestinal flora, primordial follicles, macrophages, STAT3

## Abstract

Obesity induced by a high-fat diet (HFD) leads to the excessive consumption of primordial follicles (PFs) in the ovaries. There is systemic chronic inflammation under HFD conditions, but no previous studies have explored whether there is a certain causal relationship between HFD-induced chronic inflammation and the overactivation of PFs. Here, we showed that HFD causes disorders of intestinal microflora in mice, with five Gram-negative bacteria showing the most profound increase at the genus level compared to the normal diet (ND) groups and contributes to the production of endotoxin. Endotoxin promotes M1 macrophage infiltration in the ovaries, where they exhibit proinflammatory actions by secreting cytokines IL-6, IL-8, and TNFα. These cytokines then boost the activation of PFs by activating Signal Transducer and Activator of Transcription 3 (STAT3) signaling in follicles. Interestingly, transplantation of the HFD intestinal microflora to the ND mice partly replicates ovarian macrophage infiltration, proinflammation, and the overactivation of PFs. Conversely, transplanting the ND fecal microbiota to the HFD mice can alleviate ovarian inflammation and rescue the excessive consumption of PFs. Our findings uncover a novel and critical function of gut microbes in the process of PF overactivation under HFD conditions, and may provide a new theoretical basis for the microbial treatment of patients with premature ovarian insufficiency caused by HFD.

## 1. Introduction

Premature ovarian insufficiency (POI) is one of the common causes of infertility in women of reproductive age, with an incidence of 1‰ at the age of 15–29 and 1% at the age of 30–39 [1]. The pathogenesis of POI is not yet clear, but obesity induced by a high-fat diet (HFD) has been proved to be one of the main risk factors. Accumulated studies have demonstrated that HFD can cause primordial follicle (PF) overactivation and follicular atresia, leading to the premature depletion of follicular reserves, and the eventual development of POI [2,3,4]. However, the detailed molecular mechanisms are not yet understood.

There is solid evidence that HFD-induced obesity has a significant chronic inflammatory component [5,6]. Chronic inflammation plays an important role in determining a vicious cycle with the associated pathological process of POI. For example, a large number of immune cells including macrophages were infiltrated in mouse ovaries with POI, and the levels of inflammatory cytokines were also significantly upregulated, suggesting that patients with POI are in a low-grade inflammatory state [2,7]. Additionally, in a radiotherapy-induced POI rat model, the levels of inflammatory markers and macrophage infiltration in ovarian tissue were also significantly increased [7]. These studies further illustrate the important roles of inflammation in promoting the overactivation of PFs under HFD condition.

Accumulated studies have demonstrated that the chronic inflammation induced by HFD is mediated by persistent inflammatory responses following gut microbial dysbiosis and the leakage of gut microbiota-derived endotoxin [8,9,10,11]. Disorder of the intestinal flora can disrupt the intestinal immune system and increase intestinal permeability, allowing more endotoxins to enter the bloodstream and activate the systemic immune system, thus leading to systemic metabolic inflammation and local tissue inflammation [8,12,13]. Such an intimate linkage has also been recently revealed in the male reproductive system between HFD-induced microbiota dysbiosis and the impairment of spermatogenesis with elevated endotoxin, increased intestinal infiltration of T cells and macrophages, as well as localized epididymal inflammation as the potential causes [8]. Therefore, it remains interesting to investigate whether the overactivation of PFs induced by HFD is also caused by imbalanced gut microbiota and the resulting ovarian inflammation.

In this study, we showed that HFD alters the constitution of the intestinal flora and leads to endotoxemia and ovarian macrophage infiltration, where they increase the secretion of inflammatory cytokines. Changes in the ovarian microenvironment in turn promotes the overactivation of PFs. 

## 2. Results

### 2.1. Endotoxemia Due to an Imbalance of Intestinal Microflora in HFD Mice Contributes to the Overactivation of PFs

**Figure 1:**
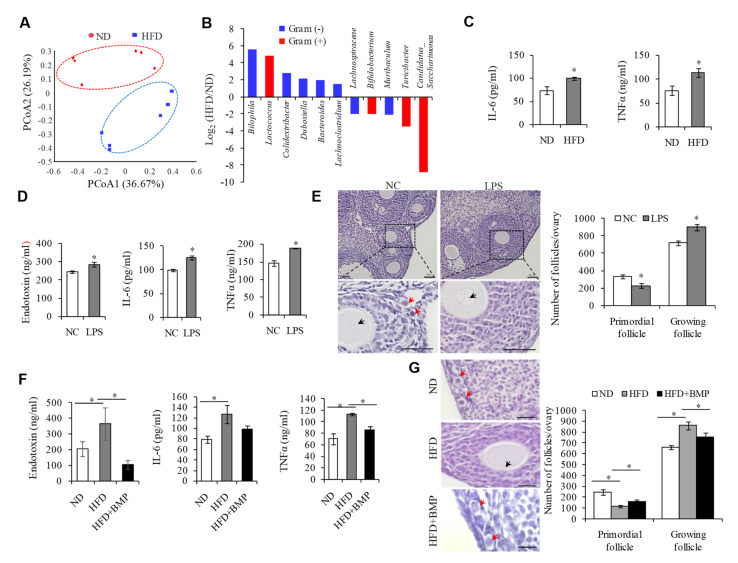
Endotoxemia due to imbalance of intestinal microflora in HFD mice contributes to the overactivation of primordial follicles. (**A**) Principal coordinate analysis (PCoA) graph based on the operational taxonomic unit (OTU) matrix of ND and HFD fecal microbiota (*n* = 6). (**B**) Log2 ratio of up- and down-regulated bacteria at the genus level between HFD and ND groups. (**C**) ELISIA detecting serum IL-6 and TNFα levels in ND (*n* = 3) and HFD (*n* = 3) mice. (**D**) ELISIA assay detecting serum concentrations of endotoxin (*n* = 5), IL-6 (*n* = 3) and TNFα (*n* = 3) protein levels in NC and LPS-treated groups. (**E**) Representative H&E staining images and follicle number counts showing that the primordial follicle pool in LPS-treated mice was significantly decreased compared to NC mice. *n* = 10. Scale bar = 100 μm. (**F**) ELISIA assay detecting serum concentrations of endotoxin, IL-6, and TNFα in ND (*n* = 3), HFD (*n* = 3), and HFD+BMP (*n* = 3) groups. (**G**) Representative H&E staining images and follicle number counts showing that blocking endotoxin with BMP could reduce the overactivation of primordial follicles when exposed HFD (*n* = 5). Red arrows represent primordial follicles. Black arrows represent growing follicles. Scale bar = 50 µm. * *p* < 0.05.

In order to examine the effect of HFD on PF activation, we first established a HFD-induced obesity mouse model, with animals fed a standard low-fat chow diet as a control (ND). Consistent with previous reports that HFD-induced obesity accelerates ovarian follicle development and follicle loss [2,3], we showed that prolonging the HFD regimen resulted in weight gain (Supplementary Figure S1A) and a significant reduction in the number of PFs (Supplementary Figure S1B). It is well-known that changes in gut microbiota control metabolic endotoxemia-induced inflammation in HFD-induced obesity in mice [14]. Our 16S-rDNA amplicon sequencing analysis of the gut microbiota between HFD and ND groups confirmed that HFD caused disorders of intestinal microflora, showing the distinct clustering of intestinal microbe communities into two groups (Figure 1A). Among the seven most significantly upregulated bacteria at the genus level compared to the ND groups, five belong to Gram-negative bacteria; namely Bilophila, Colidextribacter, Dubosiella, Bacteroides, and Lachnoclostridium. Conversely, the top two most downregulated bacteria are Gram-positive (Figure 1B), suggesting that HFD exposure elevates the proportion of Gram-negative bacteria in intestinal flora. Inflammation is believed to be closely related to the pathogenesis of POI [2,7]. Given that endotoxin is the main component of the cell wall of Gram-negative bacteria, and it is also involved in the process of the induction of the inflammatory reaction, it is possible that the elevation of endotoxin in HFD mice may be the important cause of the overactivation of PFs in HFD mice.

To test this hypothesis, we first confirmed the significant increases in inflammatory cytokines IL-6 and TNFα in the serum of the HFD mice (Figure 1C). Next, we treated the mice with lipopolysaccharide (LPS), the chemical nature of endotoxin, by intragastric administration, and found that a single infusion of LPS obviously elevated the serum concentrations of endotoxin, IL-6, and TNFα, when compared with the control mice (Figure 1D). In addition, the number of PFs was decreased, and that of growing follicles was increased upon LPS treatment (Figure 1E). We then examined whether blocking endotoxin could rescue systemic inflammation and reduce the overactivation of PFs when exposed to HFD. To test this, we treated HFD mice with Polymyxin B (BMP) by gavage, an antibiotic that kills Gram-negative bacteria and reduces the production of endotoxin, and observed that BMP not only suppressed the serum levels of endotoxin, IL-6, and TNFα (Figure 1F), but also protected against the overactivation of PFs in HFD mice (Figure 1G). These results suggested that endotoxemia due to an imbalance of the intestinal microflora can replicate the phenotype of systemic inflammation and the overactivation of PFs in HFD mice.

### 2.2. M1 Macrophages Promoted the Overactivation of PFs in HFD Mice

**Figure 2:**
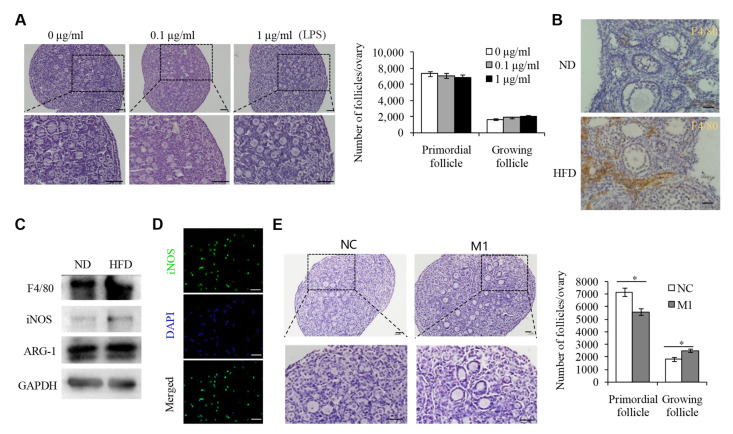
M1 macrophages promoted the overactivation of primordial follicles in HFD mice. (**A**) Representative H&E staining images and follicle number counts in the 3 dpp mouse ovaries after treatment with LPS (0, 0.1, and 1 μg/mL) for 3 days (*n* = 10). Scale bar = 50 µm. (**B**) Immunohistochemistry staining for the macrophage marker F4/80 in the ovaries of HFD mice. Scale bar = 50 µm. (**C**) WB assay detecting the protein levels of F4/80, iNOS, ARG-1, and GAPDH in the ovaries of the ND and HFD mice. (**D**) Immunofluorescence analysis detecting iNOS (green) expression in the peritoneal macrophages from LPS-treated mice. DNA was stained with DAPI (blue). Scale bar = 50 µm. (**E**) Representative H&E staining images and follicle number counts in the 3 dpp mouse ovaries supplemented with M1 macrophage compared to controls (*n* = 10). Scale bar = 50 µm. * *p* < 0.05.

To determine how endotoxin overactivates PFs in HFD mice, we collected and cultured 3 dpp mouse ovaries with a range of concentrations of LPS for 3 days and found that 0.1–1.0 μg/mL of LPS treatment did not affect the development of PFs (Figure 2A), suggesting that endotoxin may not directly regulate the overactivation of PFs. Endotoxemia could induce tissue macrophage infiltration in HFD mice [14]. Moreover, there was also strong evidence demonstrating the involvement of macrophage infiltration under ovarian inflammation, resulting in ovarian diseases including POI [15]. Consistent with this observation, our immunohistochemistry staining for the macrophage marker F4/80 clearly showed evidence of macrophage accumulation in the ovaries of HFD mice (Figure 2B). In tissues, macrophages could be activated and polarized to an M1 (pro-inflammatory) or M2 (anti-inflammatory) phenotype, depending on different extracellular milieu [16,17,18]. Endotoxin is well known as an activator of M1 type macrophages [19,20], and our results thus confirmed that, in the HFD mouse ovaries, the expression of inducible nitric oxide synthase (iNOS), an M1 macrophage marker, was upregulated, while the expression of M2 macrophage marker ARG-1 did not change (Figure 2C), implying that M1 type macrophages activated by endotoxin may play an important role in the activation of PFs in HFD mice. To this end, we collected the peritoneal macrophages (M1 type, Figure 2D) from the LPS-treated mice and co-cultured them with 3 dpp ovaries. Results showed that supplementation with M1 macrophages could obviously promote the overactivation of PFs, compared with the controls without macrophages (Figure 2E).

### 2.3. M1 Macrophage Infiltration intothe OvariesIs the Main Reason for the Overactivation of PFs in HFD Mice

**Figure 3:**
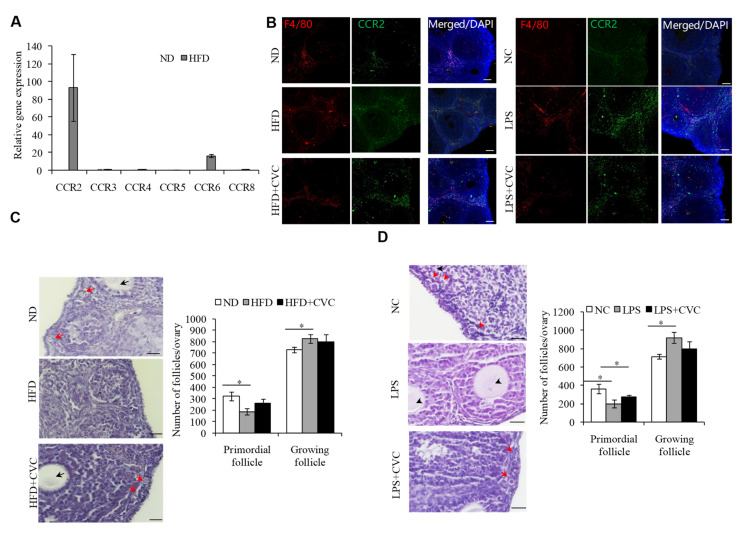
M1 macrophage infiltration into the ovaries is the main reason for the overactivation of primordial follicles in HFD mice. (**A**) qPCR analysis of the macrophage chemokine receptors in the ovaries of the ND and HFD mice. (**B**) Immunofluorescence detection of CCR2 (green) and F4/80 (red) in ND, HFD, and HFD+CVC mice (**left panel**), or in the control, LPS, and LPS+CVC treated mice (**right panel**). DNA was stained with DAPI (blue). Scale bar = 50 µm. (**C**) Representative H&E staining images and follicle number counts in the ovaries of HFD mice treated with CVC (*n* = 3), compared to the HFD mice (*n* = 5). ND mice were used as controls (*n* = 5). Scale bar = 50 μm. (**D**) Representative H&E staining images and follicle number counts in the ovaries of the control, LPS, and LPS+CVC treated mice (*n* = 5). Scale bar = 50 μm. Red arrows represent primordial follicles. Black arrows represent growing follicles. * *p* < 0.05.

Previous studies have shown that the macrophages infiltrating local tissues in HFD mice were mainly from circulating monocytes [21,22]. C-C chemokine receptor (CCR)2 can be expressed by monocytes and is required for monocyte/macrophage recruitment to sites of inflammation [23,24]. In our study, the Ccr2 mRNA level was most clearly noted in the ovaries of HFD mice, relative to the other CCR family members (Figure 3A). We therefore detected the expression levels of CCR2 to validate whether the accumulated macrophages in the ovaries of HFD or LPS-treated mice originated from circulating monocytes. As expected, the protein expression level of CCR2 was increased significantly in the ovaries of the HFD mice (Figure 3B). Conversely, we treated HFD mice with cenicriviroc (CVC), a dual CCR2/CCR5 antagonist, to inhibit the migration of macrophages from blood to the ovaries, and results showed that CVC treatment could clearly decrease the macrophage infiltration to the ovaries in the HFD or LPS-treated mice, compared to the control group (Figure 3B). Of note, inhibiting macrophage infiltration to the ovaries during HFD (Figure 3C) or LPS (Figure 3D) exposure obviously prevented HFD- or LPS-induced PF overactivation. Together, these data suggested that endotoxin, upon HFD exposure, drives macrophages infiltration into the ovaries and causes them to acquire anM1 (proinflammatory type) phenotype, where they boost ovarian inflammation and the overactivation of PFs.

### 2.4. M1 Macrophages Exhibit Proinflammatory Actions by Secreting Cytokine IL-6, IL-8, and TNFα to Promote the Overactivation of PFs

**Figure 4:**
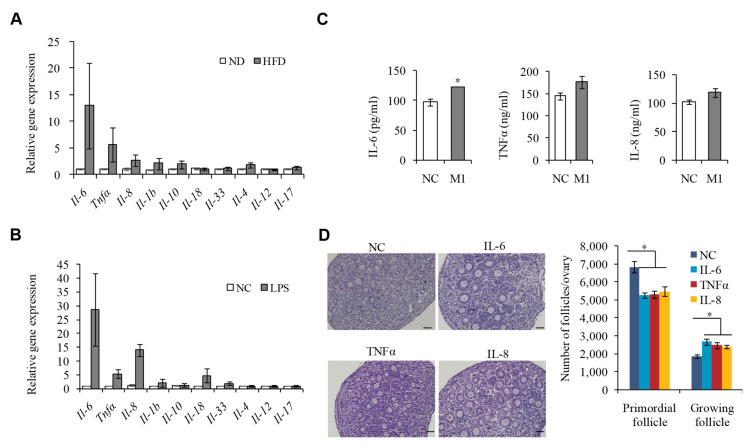
Macrophage (M1) exhibits proinflammatory actions by secreting cytokine IL-6, IL-8, and TNFα to promote the overactivation of primordial follicles. (**A**,**B**) qPCR analysis of the mRNA levels of pro-inflammatory cytokines in the ovaries of ND and HFD mice (**A**), and NC and LPS-treated mice (**B**). (**C**) ELISIA assay detecting IL-8, TNFα, and IL-6 protein levels in culture medium with or without M1 type macrophage extracted from enterocoelia of the HFD mice (*n* = 3). (**D**) Representative H&E staining images and follicle number counts in the 3 dpp mouse ovaries supplemented with IL-8, IL-6, or TNFα compared to control mice (*n* = 10). Scale bar = 50 μm. * *p* < 0.05.

Our previous immunofluorescence staining for macrophages in the ovarian sections of HFD mice has shown that macrophages were mainly localized to the theca cells, corpus luteum and atretic follicles, as well as the interstitial tissue, while not directly coming into contact with PFs, suggesting that M1 macrophages may promote inflammatory responses by secreting cytokines [15,25], unlikely via phagocytosis. To test this hypothesis, we detected the expression of a series of cytokines in the ovaries of HFD mice or LPS-treated mice compared to the control group, and Il-6, Il-8, and Tnfα were identified to be the most upregulated candidates (Figure 4A,B). Further, we measured the secretion of IL-6, IL-8, and TNFα in the conditioned media supplemented with M1 macrophages, which were extracted from enterocoelia of the HFD mice and found that the expression levels of these three cytokines were significantly elevated (Figure 4C), suggesting that IL-6, IL-8, and TNFα may be the important inflammatory cytokines that drive the overactivation of PFs under HFD. To further confirm this hypothesis, we cultured 3 dpp mouse ovaries supplemented with IL-6, IL-8, or TNFα, and found that the addition of any of IL-6, IL-8, or TNFα could partly decrease the number of PFs and increase that of growing follicles (Figure 4D). These data suggested that the M1 macrophages that infiltrate the ovaries in HFD mice may participate in the overactivation of PFs through secreting cytokines IL-8, IL-6, and TNFα.

### 2.5. IL-6, IL-8, and TNFα Promote the Overactivation of PFs in HFD Mice by Activating STAT3 Expression

**Figure 5:**
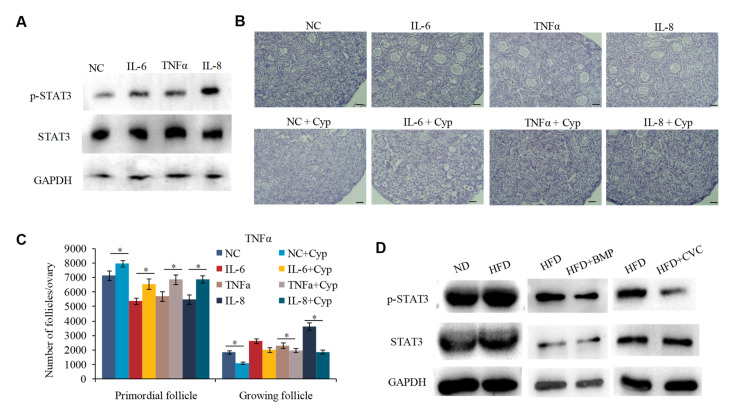
IL-6, IL-8, and TNFα promote the overactivation of primordial follicles in HFD mice by activating STAT3 expression. (**A**) WB analysis of the protein levels of p-STAT3, STAT3, and GAPDH in the ovary supplemented with IL-8 or IL-6 or TNFα. (**B**,**C**) Representative H&E staining images (**B**) and follicle number counts (**C**) in the IL-8, IL-6, or TNFα- treated ovaries supplemented with STAT3 inhibitor Cyp (*n* = 10). Scale bar = 50 μm. * *p* < 0.05. (**D**) WB analysis of the protein levels of p-STAT3, STAT3, and GAPDH in the ovaries of the ND, HFD, HFD+BMP, and HFD+CVC mice.

IL-6, IL-8, and TNFα are pleiotropic cytokines involved in inflammatory processes [26]. The activator of transcription (STAT) signaling is a highly conserved signal transduction pathway triggered by IL-6, IL-8, and TNFα [27]. It is also knownthat the activated STAT3 is crucial for maintaining the ovarian reserve of PFs in mice [28,29]. However, it is not clear whether IL-6, IL-8, and TNFα regulating PF overactivation is also triggered via the activation of STAT3 signaling. To test this, we detected the expression and phosphorylation of STAT3 in the cultured 3 dpp mouse ovaries supplemented with IL-8, IL-6, or TNFα, respectively. Immunoblotting experiments revealed that supplementation with either IL-8, IL-6, or TNFα could increase the phosphorylated STAT3 (p-STAT3) expression (Figure 5A). Moreover, blocking STAT3 with cryptotanshinone (Cyp) obviously attenuated the inductive effect of IL-6, IL-8, or TNFα on PF activation (Figure 5B,C). To further verify that the increased p-STAT3 expression was regulated by M1 macrophages in HFD mice, we treated the HFD mice with BMP to reduce the production of endotoxin, or CVC to inhibit the infiltration of macrophages to the ovaries. Results showed that HFD exposure induced STAT3 activation, but this induction was inhibited by either BMP or CVC treatment (Figure 5D). Correspondingly, the expression pattern of p-STAT3 also followed the activation state of PFs, as shown in Figure 1G and Figure 3D, under these conditions. These results suggested that the cytokines IL-6, IL-8, and TNFα may induce STAT3 activation and thus promote the overactivation of PFs in HFD mice.

### 2.6. Changing IntestinalMicroflora of HFD Mice May Correct the Ovarian Inflammation Status and the Overactivation of PFs

**Figure 6:**
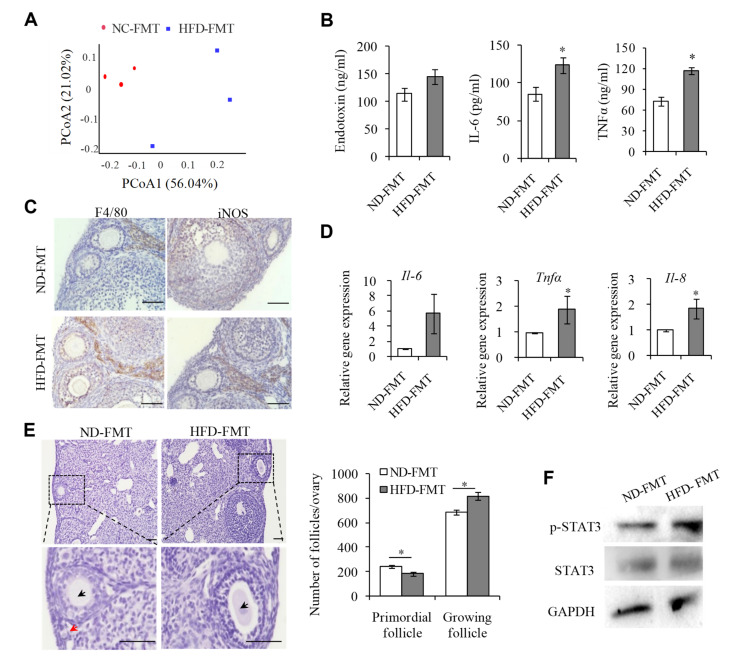
Transplantation of HFD gut microbiome leads to endotoxemia, infiltration of ovarian macrophages, and decreased primordial follicle pool. (**A**) Principal coordinate analysis (PCoA) graph based on the operational taxonomic unit (OTU) matrix of ND-FMT and HFD-FMT fecal microbiota (*n* = 3). (**B**) ELISIA analysis of serum concentration of endotoxin, IL-6, and TNFα in ND-FMT and HFD-FMT groups (*n* = 5). (**C**) Immunohistochemistry analysis of F4/80 and iNOS in ND-FMT and HFD-FMT mouse ovaries. Scale bar = 50 μm. (**D**) The mRNA levels of Il-6, Tnfα, and Il-8 in ND-FMT and HFD-FMT ovaries determined by qPCR assay. (**E**) Representative H&E staining images and follicle number counts in ND-FMT and HFD-FMT mouse ovaries. Red arrows represent primordial follicles. Black arrows represent growing follicles. * *p* < 0.05. Scale bar = 100 μm. (**F**) WB analysis of the protein levels of p-STAT3, STAT3, and GAPDH in ND-FMT and HFD-FMT mouse ovaries.

Given that we demonstrated that disorders of the intestinal microflora in HFD mice affect the PF activation status, we then hypothesized whether transferring stool samples from HFD mice to the ND mice would bring a similar phenotype to the recipients as that of the donors. The fecal microbiota transplantation (FMT) procedure was performed every 2 days, and 16S-rDNA amplicon sequencing analysis of fecal bacteria revealed that the gut microbial ecosystem in the recipient mice was disturbed 8 weeks after transplantation of the fecal bacteria from the HFD mice (HFD-FMT), compared to those receiving the fecal bacteria from the ND mice (ND-FMT) (Figure 6A). Interestingly, we also identified Dubosiella and Colidextribacter, two bacteria at the genus level showing obviously increased abundances, in line with the changes in HFD mice, indicating that the intestinal flora of HFD mice could be colonized in the intestines of HFD-FMT mice. Next, we investigated whether the HFD-FMT mice could replicate the phenotypes of ovarian inflammation and PF overactivation observed in HFD mice. Results showed that the serum levels of endotoxin, IL-6, and TNFα in HFD-FMT mice were significantly up-regulated compared with those of control ND-FMT mice (Figure 6B). Meanwhile, we observed an increased number of M1 macrophages in the ovaries of HFD-FMT mice (Figure 6C), and the expression of Il-6, Il-8, and Tnfα was also significantly upregulated in the ovaries of HFD-FMT mice (Figure 6D). Correspondingly, the ovarian reserve of PFs in HFD-FMT mice was reduced (Figure 6E), accompanied by the activation of STAT3 (Figure 6F).

**Figure 7:**
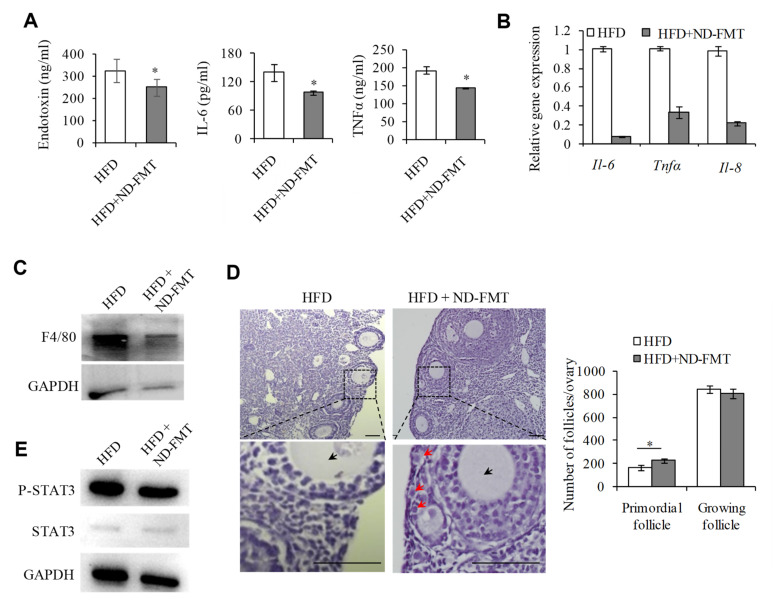
Transplantation of normal mice intestinal flora could rescue HFD induced overactivation of primordial follicles. (**A**) ELISIA analysis of serum concentration of LPS, TNFα, and IL-6 protein levels in HFD and HFD+ND-FMT groups (*n* = 4). (**B**) The mRNA levels of Il-6, Tnfα, and Il-8 in HFD and HFD+ND-FMT mouse ovaries determined by qPCR assay. (**C**) WB analysis of the protein levels F4/80 in HFD and HFD+ND-FMT mouse ovaries. GAPDH used as a loading control. (**D**) Representative H&E staining images and follicle number counts in HFD and HFD+ND-FMT mice (*n* = 5). Red arrows represent primordial follicles. Black arrows represent growing follicles. Scale bar = 100 μm.* *p* < 0.05. (**E**). WB analysis of the protein levels of p-STAT3, STAT3, and GAPDH in HFD and HFD+ND-FMT mouse ovaries. (**F**) Proposed model for intestinal flora changes induced by a HFD exacerbating activation of primordial follicles through macrophage infiltration and inflammatory factor secretion in mouse ovaries.

Conversely, we considered whether transplanting the intestinal flora from ND mice to HFD mice could alleviate the ovarian inflammation and the PF overactivation in the recipient mice. As shown in Figure 7A, this treatment resulted in decreases in serum endotoxin, IL-6, and TNFα, and the expression of these cytokines was also decreased in the ovaries of the HFD mice receiving the intestinal flora from the ND mice (HFD+ND-FMT), compared with those of HFD mice (Figure 7B). In line with these changes, the number of M1 macrophages in the ovaries of the recipient HFD mice receiving the intestinal flora from the ND mice was also reduced (Figure 7C), accompanied by an increased quantity of PFs (Figure 7D). Finally, the phosphorylated STAT3 was significantly down-regulated in the recipient HDF mice receiving the intestinal flora from ND mice (Figure 7E). Taken together, our findings indicated that the dysbiosis of gut microbiota induced by HFD could result in ovarian inflammation and the overactivation of PFs.

## 3. Discussion

**Figure 8:**
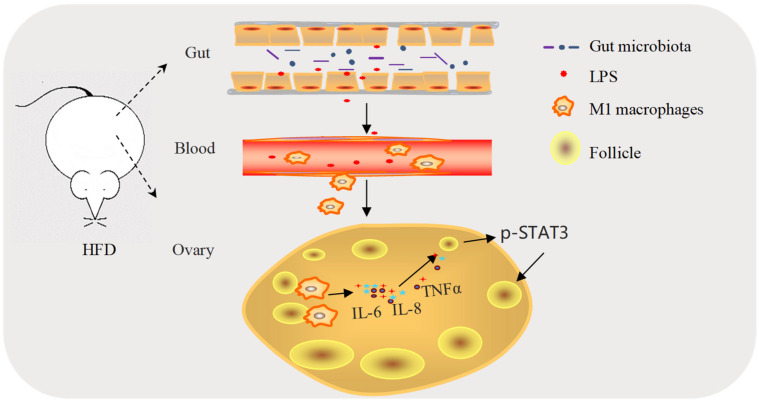
Proposed model for intestinal flora changes induced by a HFD exacerbating activation of primordial follicles through macrophage infiltration and inflammatory factor secretion in mouse ovaries.

As a common cause of female infertility, POI is a multifactorial disease, in which HFD represents one of the most important factors [3,30]. Meanwhile, it is well accepted that HFD-induced gut dysbiosis is the origin to promote low-grade systemic chronic inflammation [2,9,10,11]. However, it is not clear whether HFD-induced chronic inflammation is responsible for the excessive consumption of PFs in ovaries, and ultimately triggers the development of POI. Interestingly, a recent study showing that the reduction in PFs and the compromised fertility under HFD exposure in mice are accompanied by higher proinflammatory cytokine levels and increased ovarian macrophage infiltration further supports the possibility that the detrimental effect of HFD on PFs may be mediated by increased ovarian tissue inflammation [2]. On the premise of these findings, our study has taken one step forward and clarified in detail that the imbalance of the intestinal microflora induced by HFD may be one of the main reasons for the accelerated consumption and eventually the depletion of the PF pool. Mechanistically, the HFD-induced upregulation of Gram-negative bacteria in the gut microbiota leads to the increased blood endotoxin, which promotes M1 macrophages’ infiltration in the ovaries and induces M1 macrophages to secrete cytokines IL-6, IL-8, and TNFα. These cytokines then boost the activation of PFs by activating STAT3 signaling in oocytes (Figure 8). Our findings establish a functional link between HFD-induced intestinal microflora disorders and the excessive consumption of PFs in ovaries, leading to a decreased PF reserve.

As for the possible causes of HFD-induced inflammation, recent studies have shown that the imbalance of intestinal flora induced by HFD is the main inducement of chronic, and low-grade inflammation in obese patients [21], and the dysbiosis of gut microbiota induced by HFD has been attributed to the occurrence of reproductive system diseases. For example, individuals with polycystic ovary syndrome (PCOS) have gut microbiota communities different from those of healthy controls [31,32,33]. HFD-induced imbalanced gut microbiota was shown to be one of the primary causes for the impairment of spermatogenesis, likely mediated by elevated blood endotoxin, epididymal inflammation, and the dysregulation of testicular gene expressions [8].Transferring the gut microbiota from obese subjects into germ-free mice partially replicated the increased body weight and other chronic inflammatory diseases in the obese subjects [34,35,36], further demonstrating a causality between the imbalanced microbiota and metabolic diseases caused by HFD [34,36,37].Consistent with these findings, we also identified the upregulated Gram-negative bacteria from the intestinal microflora in HFD mice compared to the control group, which contributes to low-level endotoxemia. Interestingly, the population increases of Dubosiella and Colidextribacter in the HFD-FMT mice reflect similar increases of those in the HFD mice, indicating the successful colonization of these Gram-negative bacteria in the intestine of transplant recipients. Importantly, our fecal microbial transplantation manipulation confirmed that the modulation of the gutmicrobiota is responsible for the ovarian inflammatory state and the overactivation of PFs in HFD mice, thus providing a rationale for restoring the gut microbial ecosystem under HFD conditions to alleviate the overactivation of PFs and protect the ovarian follicle reserve.

Though previous studies have showed that endotoxin can deplete the PFs pool [20,38,39], it is not clear how endotoxin exerts this critical impact. In this study, we have provided the evidence to demonstrate that HFD-induced endotoxemia promotes M1 macrophage infiltration in the ovaries, where they stimulate the overactivation of PFs by secreting cytokines and activating STAT3 signaling in the oocytes. Given that CCR2 expression on monocytes is required for monocyte/macrophage recruitment to the inflammatory loci [23,24], we treated the HFD mice with the CCR2 antagonist CVC to block the recruitment of monocyte-derived macrophages from the blood to the ovaries and then confirmed that the inhibition of M1 macrophages infiltration has protective effects against HFD-induced macrophages recruitment and the overactivation of PFs. Of note, CCR2 is not unique to monocytes/macrophages; therefore, the in hibition of CCR2 may also affect the migration of other types of inflammatory cells [40]. To more directly answer this question, we established a co-culture system of macrophages and ovarian tissues, and showed that endotoxin-treated macrophages (M1 type) could promote the overactivation of PFs. Thus, our results provide insights into the role of M1 macrophages in the regulation of the ovarian inflammation state, and refine the potential molecular mechanism of endotoxin-mediated PF activation induced by HFD. 

Macrophages are capable of releasing cytokines, chemokines, and growth factors that play essential roles in both physiological and pathological conditions [41], including many aspects of ovarian function [2,15,42,43,44]. Given that the expression and activity of proinflammatory factors TNFα, IL-6, and IL-8 have frequently been reported in the ovaries of obese patients [29,45,46], it is reasonable to observe that the M1 macrophages that infiltrate the ovaries may participate in the regulation of PF activation via these proinflammatory cytokines. Furthermore, it is known that proinflammatory cytokines such as IL-6 [47], TNFα [48], and IL-8 [26] can activate the downstream STAT3 signaling in tumors. We showed here, for the first time, that these three cytokines can also activate STAT3 in oocytes. The activated STAT3 then translocates to the nuclei of the oocytes, where they activate the transcription of essential oocyte-specific genes, and positively regulate PF activation in mice [29,49,50]. Our results are in good agreement with these observations and strongly suggest that M1 macrophages that infiltrate the ovaries of HFD mice may secrete L-6, IL-8, and TNFα, which then participate in the regulation of PF activation by activating STAT3 signaling.

Overall, our findings established that HFD-induced gut dysbiosis and the release of endotoxin are the primary causes of systemic chronic inflammation with M1 macrophage infiltration in the ovaries, where they secrete proinflammatory cytokines to stimulate the overactivation of PFs and thus reduce the ovarian follicle reserve. Our results provide conceptually novel insights into the pathogenesis of POI under HFD conditions by uncovering the role of the gut microbiota in shaping the immune response, and may also offer therapeutic implications for restoring the gut microbial ecosystem to improve ovarian function.

## 4. Materials and Methods

### 4.1. Animal Study

All the C57BL/6J mice were purchased from the Animal Core Facility of Nanjing Medical University. The Animal Care and Use Committee of Nanjing Medical University approved all the animal experiments (IACUC-2004024, 24 April 2020). The mice were housed under a 12/12-h dark/light cycle with free access to food and water at 20–22 °C. The 4-week-old mice were randomly divided into normal diet (ND), high fat diet (HFD), ND faecal microbiota transplantation (ND-FMT), and HFD faecal microbiota transplantation (HFD-FMT) groups and HFD mice receiving ND faecal microbiota transplantation (HFD+ND-FMT) groups (5 mice per group). The ND group and the HFD group were fed with an ND or an HFD (Research Diet D12492, 60% fat), respectively. The mice were sacrificed after 12 weeks of dietary treatment for the harvesting of serum and tissues. 

In the LPS-treatment study, the mice were given a 5 mg/kg dose of LPS (from *E. coli* 0111:B4) solution (0.9% NaCl solution as solvent) by gavage every 5 days as described previously [51], while the same dose of 0.9% NaCl solution was given to mice in the NC group. Mice were sacrificed after the second treatment for the harvesting of serum and tissues; In the Polymyxin B (BMP) treatment study, the HFD mice were then randomly selected for 5 mg/kg BMP treatment by intragastric administration every 2 days [51]. The intervention lasted 8 weeks. In the cenicriviroc (CVC) treatment study, the mice received either a placebo control (vehicle) or a CVC (15 mg/kg body weight) subcutaneous injection daily as described previously [40], starting from the day before the LPS treatment and lasting 9 days thereafter; In the HFD and CVC treatment studies, the mice pretreated with HFD for 4 weeks received either 0.9% NaCl or 15 mg/kg CVC every 2 days for an additional 2 weeks before sacrifice. 

In the FMT study, 0.2 g of fresh stool samples was collected from the ND or HFD mice immediately on defecation and resuspended in 4 mL of saline, vortexed for 5 min, and filtered with sterile gauze. The transplantation into the recipient mice was achieved by gavage with 200 µL of the supernatant from the faecal sample once every 2 days and supplied with ND for 12 weeks. In the rescue experiment of FMT, the mice pretreated with HFD were orally gavaged with fecal suspension from ND or HFD mice, and supplied with HFD for 12 weeks.

### 4.2. Ovary Culture

The mice were mated using timed mating, and the presence of a vaginal plug was defined as 0.5day post coitus (dpc). Neonatal ovaries were collected at 3 dpp. Four ovaries were placed in a single well of a 24-well dish and cultured in α-minimal essential medium (α-MEM, Cat# 12571063, Invitrogen, Carlsbad, CA, USA) supplemented with 5% FBS, 100 IU/mL penicillin, and 100 μg/mL streptomycin at 37 °C under 5% CO2. Where indicated, the 3 dpp ovaries were treated with 0.1, 1.0 μg/mL of LPS (Cat# O111: B4, Sigma, St. Louis, MI, USA), or the recombinant IL-6, TNFα, and IL-8 (50 ng/mL, 50 ng/mL, and 50 ng/mL, respectively) for 3 days. The same volume of PBS was used as a control. 

### 4.3. Quantitative Real-Time PCR

RNA was extracted from mouse ovaries using TRizol (Cat# 15596026, Invitrogen, Carlsbad, CA, USA), followed by reverse transcription with First Strand cDNA Synthesis Kit (K1621, Thermo Scientific, Waltham, MA, USA), and qRT-PCR analysis with AceQ qPCR SYBR Green Master Mix (Cat# Q141-02, Vazyme, Nanjing, China). The primers are summarized in Supplementary Table S1. The relative fold change of gene expression was calculated using the relative standard curve method (2^−ΔΔCt^). 

### 4.4. Western Blotting

Ovaries were lysed with radioimmunoprecipitation buffer (Cat# CW2333, CWBIO, Beijing, China) with 1× protease inhibitor (Cat# CW2200, CWBIO, Beijing, China). The supernatant was collected, followed by BCA protein assay for determination of protein concentration. Approximately30 μg of denatured protein was separated on a 10% SDS-PAGE gel, and transferred to a PVDF membrane. The blots were rinsed in TBS containing 0.1% Tween-20, and blocked with 5% nonfat dry milk. After incubation with the primary antibodies including anti-F4/80 (Cat# ab6640, Abcam, Cambridge, UK), anti-iNOS (Cat# ab49999, Abcam, Cambridge, UK), anti-STAT3 (Cat# 60199, Proteintech, Hubei, China), anti-p-STAT3 (Cat# 9145, Cell signaling, Massachusetts, Danvers, MA, USA), anti-ARG-1 (Cat# ab91279, Abcam, Cambridge, UK), and anti-GAPDH (Cat# ab8245, Abcam, Cambridge, UK) overnight at 4 °C, the blots were then washed and incubated with corresponding peroxidase-conjugated secondary antibody for 1h at room temperature. The signals were visualized using an Enhanced Chemiluminescence Detection Kit (Cat# 32106, Thermo Scientific, Waltham, MA, USA) on a Bio-Rad gel imaging system. For p-STAT3 and STAT3 antibodies, they were stripped and re-probed sequentially. For other antibodies, the images composite from different blots.

### 4.5. Immunofluorescence

The mouse ovary tissue sections were deparaffinized and rehydrated, followed by antigen retrieval by boiling the sections in 0.01 M citrate buffer, pH 6.0 for 15 min. Then the sections were blocked in 10% normal goat serum, and incubated with primary antibodies including anti-CCR2 (Cat# DF7507, Affinity Biosciences, Changzhou, China), anti-F4/80 (Cat# ab6640, Abcam, Cambridge, UK), and anti-iNOS (Cat# ab49999, Abcam, Cambridge, UK) overnight at 4 °C. The mouse, rat, or rabbit IgG was used as primary antibody negative control. After 5 washes with PBS, the sections were incubated with secondary antibodies for 1 h at room temperature. DAPI was used to stain nuclei. The sections were examined under a confocal laser scanning microscope (LSM 700; Zeiss, Oberkochen, Germany).

### 4.6. Follicle Counting

The mouse ovaries were removed and fixed in formalin for 24 h, dehydrated in graded ethanol, embedded in paraffin and sectioned (4 µm in thicknesses). The sections were stained with H&E dye and observed under a light microscope (Leica DM500, Leica Microsystems, Wetzlar, Germany). After staining, follicles were counted every six slices. Germ cells not surrounded by germ cells were scored as unassembled cysts. Germ cells surrounded by a single layer of flattened granulosa cells or a mixture of flattened and cuboidal granulosa cells were scored as PFs. Germ cells surrounded by one or more layers of cubical granulose cells were scored as growing follicles. 

### 4.7. Isolation of Macrophages

The peritoneal eluent was collected following intraperitoneal injection of cold PBS by centrifugation at 1500 rpm for 5 min. The cell pellet was re-suspended in DMEM supplemented with 5% FBS, 100 IU/mL penicillin, 100 μg/mL streptomycin and seeded in 6 well plates. After 20 min, the wells were washed twice with PBS to remove the non-adherent cells and incubated overnight at 37 °C and 5% CO₂.

### 4.8. Immunohistochemistry

Ovaries were collected and fixed in formalin for paraffin embedding and sectioning. The sections were then deparaffinized and rehydrated. Endogenous peroxidase activity was blocked by incubating the sections in 3% hydrogen peroxide in methanol for 15 min. The sections were then boiled in 0.01 M citrate buffer to retrieve the antigen. After blocking by 10% goat serum for 1 h, the primary antibodies including anti-F4/80 (Cat# ab6640, Abcam, Cambridge, UK) and anti-iNOS (Cat# ab49999, Abcam, Cambridge, UK) were added and incubated overnight at 4 °C Therator mouse IgG was used as primary antibody negative control. Next, the sections were incubated for 1h with biotinylated secondary antibodies at 37 °C. Finally, the reaction was visualized with diaminobenzidine solution and photographed using LEICA DM2500 microscope with a LEICA DMC6200 camera.

### 4.9. Enzyme-Linked Immunosorbant Assay (ELISA) 

Mouse LPS, IL-6, IL-8, and TNFα (Cat# D5060, Cat# D8000C, Cat# DCP00, Cat# DM3A00; R&D Systems, Minneapolis, MN, USA) in the supernatants were measured using ELISA kits following the manufacturer’s instructions. 

### 4.10. 16.S rDNA High-Throughput Sequencing and Analysis

Total fecal bacteria DNA was extracted using the CTAB/SDS method. The V3-V4 hypervariable region of the 16S rDNA was amplified using a universal forward sequencing primer and a uniquely barcoded reverse sequencing primer. Sequencing libraries were generated using TruSeq^®^ DNA PCR-Free Sample Preparation Kit (Illumina, San Diego, CA, USA), following the manufacturer’s recommendations. The library quality was assessed on the Qubit 2.0 Fluorometer (Thermo Scientific) and Agilent Bioanalyzer 2100 system. The library was sequenced on an Illumina NovaSeq platform. Paired-end reads were merged using FLASH (VI.2.7, http://ccb.jhu.edu/software/FLASH/, accessed on 26 May 2021). Quality filtering on the raw tags was performed under specific filtering conditions to obtain the high-quality clean tag according to the QIIME (V1.9.1, http://qiime.org/scripts/split libraries fastq.html, accessed on 26 May 2021) quality-controlled process. The tags were compared with the reference database using the UCHIME algorithm (UCHIME Algorithm, http://www.drive5.com/usearch/manual/uchime_algo.html, accessed on 26 May 2021) to detect chimera sequences. The chimera sequences were removed, and the effective tags were finally obtained.

### 4.11. Statistical Analysis

All experiments were repeated in at least three independent biological replicates and results were presented as the mean ± the standard error of the mean. Statistical comparison between two measurements was analyzed by t-test, or one-way ANOVA, accordingly. When analyzing gut microbiota sequencing data, we used Metastats software to test the hypothesis of species abundance between groups. A value of *p* < 0.05 was considered statistically significant.

## 5. Conclusions

Our findings uncover a novel and critical function of gut microbes in the process of PF overactivation under HFD conditions, and may provide a new theoretical basis for the microbial treatment of patients with POI caused by HFD.

## Data Availability

Data sharing is not applicable to this article as no datasets were generated or analyzed during the current study.

## Supplementary Materials

The following supporting information can be downloaded at: https://www.mdpi.com/article/10.3390/ijms23094797/s1.
