# Supplementary material for: Intestinal Flora Changes Induced by a High-Fat Diet Promote Activation of Primordial Follicles through Macrophage Infiltration and Inflammatory Factor Secretion in Mouse Ovaries
Source: Int J Mol Sci. 2022 Apr 27;23(9):4797. doi: 10.3390/ijms23094797 (PMC9100959; doi:10.3390/ijms23094797)
Supplement: Supplementary file 1 [file ijms-23-04797-s001.zip › ijms-1659605-supplementary.pdf]

International Journal of
Molecular Sciences

MDPI

Intestinal Flora Changes Induced by a High-Fat Diet Promote Activation of Primordial Follicles through Macrophage Infiltration and Inflammatory Factor Secretion in Mouse Ovaries

Supplementary Figure and Figure Legend

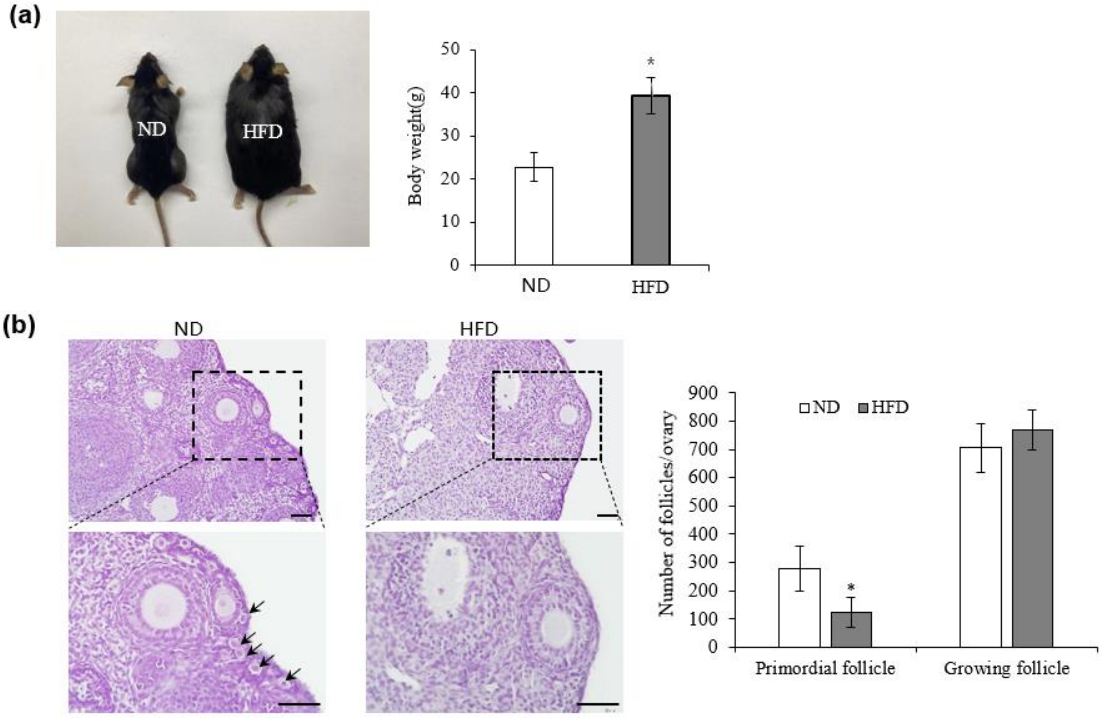

Figure S1. HFD induces the overactivation of primordial follicles in an HFD-induced obesity mouse model. (**a**) Average body weight after 12 weeks of ND (*n* = 19) or HFD diet (*n* = 20). (**b**) Representative H&E staining images and follicle number counts showing that the primordial follicle pool in HFD mice was significantly decreased compared to ND mice. Black arrows represent the primordial follicles. Scale bar = 100μm. * $P < 0.05$.

Table S1. Primer sets used in this study.

Gene (Mouse)	Primer Sequences (5′–3′)
TNFα	F: 5′-CCGATGGGTTGTACCTTGTC
	R: TGGAAGACTCCTCCCAGGTA
Il-6	F: 5′-TGCAAGAGACTTCCATCCAG
	R: 5′-TCCACGATTTCCCAGAGAAC
Il-8	F: 5′-ACTCCAAACCTTTCCACCCC
	R: 5′-TTCTCAGCCCTCTTCAAAAACTTC
Il-1b	F: 5′-GCAACTGTTCCTGAACTCAACT
	R: 5′-ATCTTTTGGGGTCCGTCAACT
Il-18	F: 5′-AAGAAAGCCGCCTCAAACCT
	R: 5′-AGTGAAGTCGGCCAAAGTTGT
Il-10	F: 5′-AGGATGCACATCAAAAGGCTT
	R: 5′-GGCCTCGGTTAGGAAGGATAC

Il-12	F: 5′-CAATCACGCTACCTCCTCTTTT
	R: 5′-CAGCAGTGCAGGAATAATGTTTC
Il-4	F: 5′-CCTCACAGCAACGAAGAACA
	R: 5′-TGGACTCATTCATGGTGCAG
Il-33	F: 5′-ACTGCATGAGACTCCGTTCTG
	R: 5′-CCTAGAATCCCGTGGATAGGC
Il-17	F: 5′-GAAGGCAGGAATCACAATC
	R: 5′-GCCTCCCAGATCACAGA
Ary-1	F: 5′-AACACTCCCCTGACAACCAG
	R: 5′-GCAAGCCAATGTACACGATG
Gapdh	F: 5′-AGGTTGTCTCCTGCGACTTCA
	R: 5′-GGGTGGTCCAGGGTTTCTTACT
Il-8	F: 5′-ACTCCAAACCTTTCCACCCC
	R: 5′-TTCTCAGCCCTCTTCAAAAACTTC
Ccr2	F: 5′-TAAAAAACCTGGATCGGAACCAA
	R: 5′-GCATTAGCTTCAGATTTACGGGT
Ccr3	F: 5′-AACTTGCAAAACCTGAGAAGC
	R: 5′-ACCATCATGTTGCCCAGGAG
Ccr4	F: 5′-GCCAACAGCCCTGTTTTCTG
	R: 5′-ACACTGGATTTGAGGCTCCG
Ccr5	F: 5′-ACTGCTGCCTAAACCCTGTC
	R: 5′-AGTGGTTCTTCCCTGTTGGC
Ccr6	F: 5′-CCCGTCTCTCAATGAGCACT
	R: 5′-AACACGAGAACCACAGCGAT
Ccr8	F: 5′-ACGTCACGATGACCGACTACT
	R: 5′-CCCAGCACAAACAAGACGC
